# From Prediction to Prevention: Using Text Mining and Explainable Machine Learning for Urban Bus Accident Analytics

**DOI:** 10.1111/risa.70183

**Published:** 2026-01-30

**Authors:** Bowei Chen, Yufei Huang, Yu Zheng, Xiaofeng Liu

**Affiliations:** ^1^ Adam Smith Business School University of Glasgow Glasgow UK; ^2^ Trinity Business School Trinity College Dublin Dublin Ireland; ^3^ School of Finance Southwestern University of Finance and Economics Chengdu China; ^4^ College of Artificial Intelligence and Automation Hohai University Nanjing China

**Keywords:** bus accident analytics, explainable machine learning, SHapley Additive exPlanations, topic modeling

## Abstract

Urban bus accidents present major safety and operational challenges, particularly in densely populated metropolitan areas. This study develops a machine learning‐based analytical framework to identify, quantify, and interpret the factors associated with severe bus accidents. The framework integrates three components: (i) a structural topic model (STM) to extract latent accident scenarios from unstructured narrative data, (ii) an extreme gradient boosting (XGBoost) classifier to predict accident severity, and (iii) SHapley Additive exPlanations (SHAP) for post hoc interpretation of model outputs at both global and local levels. Using over 15,000 bus accident records (2013–2018) from a Tier‐2 city in Jiangsu Province, China, the findings show that incorporating text‐derived accident patterns markedly improves both predictive accuracy and interpretability. The analysis highlights elevated risks linked to rear‐end collisions involving electric scooters, sudden stops leading to passenger injuries, and left‐turn maneuvers in congested areas. SHAP‐based explanations yield actionable insights for drivers, transit operators, and policymakers, facilitating targeted safety interventions. Methodologically, this study advances interpretable risk modeling through the integration of structured and unstructured data, and the modular analytical framework provides a transferable foundation for applications across diverse domains of transportation and risk analysis.

## 1 Introduction

Urban transportation safety has become as a critical issue in contemporary risk management as cities worldwide confront increasing traffic density, multimodal network complexity, and sustainability pressures. Road traffic accidents remain one of the leading causes of mortality globally, accounting for approximately 1.19 million deaths each year (World Health Organization 2023). The burden of these incidents falls disproportionately on urban areas in low‐ and middle‐income countries, where rapid motorization has outpaced safety regulation and infrastructure development (Ehsani et al. 2023). Within this broader context, bus accidents represent a distinct and consequential risk domain. Although public transport is generally recognized as a safer and more sustainable mode of travel than private vehicles, the large passenger capacity of buses amplifies the social and economic impact of each incident, often resulting in multiple casualties, operational disruptions, and diminished public confidence in the transportation system (United Nations Economic Commission for Europe 2015; Beck et al. 2007; Savage 2013; Morency et al. 2018). As cities increasingly prioritize bus‐based networks to address environmental and congestion challenges (United Nations 2022), understanding and mitigating the determinants of bus accident risk have become essential to achieving both sustainable mobility and resilient urban infrastructure. These challenges underscore the need for advanced analytical frameworks capable of capturing the multifactorial, nonlinear, and context‐dependent nature of bus accident risk to inform evidence‐based prevention and strategic decision‐making.

Prior studies on transportation safety have predominantly relied on traditional statistical approaches to identify the determinants of accident frequency and severity. While these models have generated valuable insights, they are constrained by assumptions of linearity, independence, and data normality, and typically depend on structured variables derived from standardized accident reports or numerically encoded survey and interview data. Such limitations restrict their ability to capture the nonlinear, multifactorial, and context‐dependent dynamics of accident causation, where human behavior, environmental conditions, and operational factors interact in complex ways. With the increasing availability of large‐scale unstructured data‐such as narrative accident descriptions, incident reports, and sensor logs‐recent advances in text mining and machine learning provide new opportunities to address these challenges. By integrating structured and unstructured data, these techniques can uncover latent contextual and behavioral patterns embedded in accident narratives and develop flexible predictive models capable of representing complex nonlinear relationships. Building on these developments, this study proposes an interpretable machine learning framework that combines topic modeling, predictive analytics, and explainable machine learning (or explainable AI) techniques. This unified approach advances the methodological frontier of transportation risk analysis by enhancing predictive accuracy, improving interpretability, and generating actionable insights to inform evidence‐based policy design, driver training, and operational safety management.

In this research, we introduce an analytical framework for bus accident risk analytics that comprises three progressive stages: (i) a topic modeling that uncovers latent semantic structures within unstructured accident text descriptions and performs automatic feature discovery and engineering; (ii) a predictive modeling that ensures high accuracy in forecasting accident severity; and (iii) a post hoc explanation that offers both local and global interpretability of model predictions. These stages are operationalized through the use of three popular machine learning techniques: structural topic modeling (STM), extreme gradient boosting (XGBoost), and SHapley Additive exPlanations (SHAP), respectively. Each selected for its balance of effectiveness, computational efficiency, and interpretability. To validate the framework, we conduct empirical analysis using a comprehensive bus accident dataset from a Tier‐2 city in China, which includes both structured variables and unstructured textual narratives. The results demonstrate strong performance in terms of both predictive accuracy and interpretability. STM identifies 15 coherent and semantically meaningful patterns from accident narratives, enriching the feature space and revealing distinctive contextual mechanisms of accident occurrence. The fine‐tuned XGBoost model outperforms multiple benchmark classifiers, while the SHAP analysis highlights many STM‐derived patterns as key contributors to severity prediction. Collectively, these findings validate the methodological efficacy of the proposed framework and provide novel insights to inform targeted, data‐driven strategies for accident prevention and urban transport safety management.

This study makes both methodological and empirical contributions to the field of risk analysis. Methodologically, it proposes a novel explainable machine learning framework that balances predictive power with interpretability. The framework operates in three stages: extracting latent features from unstructured accident narratives via topic modeling, predicting accident severity using a nonlinear classifier, and interpreting model outputs through post hoc explanation. Its modular and extensible design allows for flexible substitution of methods at each stage, supporting its application across diverse datasets and analytical contexts. Empirically, this work is among the first to analyze bus accidents using a large‐scale dataset that integrates structured variables with unstructured narrative descriptions. This comprehensive dataset enables a nuanced examination of accident risk factors across driver behavior, vehicle characteristics, environmental conditions, and organizational practices. The analysis identifies several high‐risk scenarios, such as sudden stops leading to passenger injuries, rear end collisions with electric scooters, and complex left hand turns, which represent critical intervention points. These findings provide actionable insights at multiple levels: promoting anticipatory driving and route familiarity among drivers; enhancing safety training and performance monitoring for operators; and informing infrastructure design, regulatory policy, and public awareness initiatives for policymakers.

The remainder of this paper is organized as follows: Section 2 provides a review of the existing literature on traffic accident analytics, with particular emphasis on current methodological approaches on bus accident studies and gaps that motivate our study. Section 3 details the proposed analytical framework, explaining its three‐stage architecture and the rationale for the selected machine learning techniques. Section 4 describes the dataset, outlines the experimental details, and presents the analysis of results. Section 5 highlights the study's methodological and empirical contributions, synthesizing key findings and exploring their implications for accident prevention from the perspectives of drivers, transit operators, and policymakers. Finally, Section 6 concludes the paper and suggests directions for future research. For clarity and focus, additional technical details and extended empirical results are presented in the Supporting Information.

## 2 Literature Review

Traffic accident studies focus on accident prediction, severity classification, and risk inference. They typically model either accident frequency or severity as the dependent (or target) variable. *Accident frequency* refers to the number of traffic accidents occurring within a specific location or time period and is usually represented as a count variable. *Accident severity*, on the other hand, describes the outcome level of an accident, such as fatal, serious injury, vehicle or property damage, and is commonly represented as a binary or ordinal variable. These target variables are explained by a wide range of factors, including driver characteristics, vehicle attributes, roadway design, environmental conditions, and other operational factors.

Various types of accidents, including those involving private vehicles, trucks, motorcycles, and buses, have been extensively investigated, with hundreds of studies employing diverse analytical methods and datasets. Recent reviews on accident studies highlight the increasing sophistication of analytical methods, from classical regression and hierarchical models to data mining and machine learning approaches. Early traffic safety research relied primarily on regression‐based statistics models such as Linear Regression (Cafiso et al. 2010), Logistic Regression (Blower and Green 2010; Rahman et al. 2011; Ghoubaira et al. 2021), Ordered Logit or Probit (Chang and Mannering 1998; Anastasopoulos and Mannering 2011; J. Li et al. 2024), Poisson (Aguero‐Valverde and Jovanis 2008;;2009; Chiou and Fu 2013), and Negative Binomial models (Jones and Jørgensen 2003; Xu and Huang 2015; X. Wang et al. 2024). These approaches were favored for their interpretability and suitability for structured and small‐to‐medium datasets. While traditional models have advanced the quantitative understanding of traffic accidents, their linearity and independence assumptions limit their ability to capture nonlinear and high‐order interactions among multiple contributing factors. The increasing availability of large‐scale, high‐dimensional datasets has driven a methodological shift toward data mining and machine learning approaches. Techniques such as Decision Trees (Chong et al. 2005; Chang and Wang 2006; Chang and Chien 2013), Random Forests (X. Li et al. 2018; Zeng et al. 2019), Support Vector Machines (Gu et al. 2018; Hadjidimitriou et al. 2020; Mokhtarimousavi et al. 2019; Xi et al. 2019), and Neural Networks (Mussone et al. 2017; Sameen and Pradhan 2017; Gu et al. 2018; Z. Zheng et al. 2019; Hadjidimitriou et al. 2020) can model nonlinear and interactive effects without strict parametric constraints. Due to space constraints, the following discussion focuses on methodological developments most relevant to our study, comprehensive reviews are provided by Lord and Mannering (2010), Silva et al. (2020), Wen et al. (2021), Mannering et al. (2021), and Skaug et al. (2025).

Bus accident analytics is a niche yet increasingly important field, reflecting the distinct operational and societal characteristics of public transport. Compared with private vehicles, bus accidents differ not only in scale and consequence but also in institutional and operational context. Although buses operate at lower accident frequencies per passenger‐kilometer, their high occupancy means that a single incident can result in multiple injuries, severe service disruption, and loss of public confidence. Furthermore, organizational factors introduce additional layers of risk and variability across transport systems. Most prior studies focused on bus accidents draw on relatively small or region‐specific samples and differ considerably in their inclusion of institutional and operational variables (Nguyen and Nguyen 2022). To enhance comparability and generalizability across research contexts, Table 1 summarizes existing bus accident studies according to five contributory categories (i.e., driver, vehicle, road, institutional, and other) and their methodological approaches.

**TABLE 1:** Summary of key bus accident studies and our research.

				Variable						
		Number of accidents			Independent/feature^a^					
Literature	Period	(Data size)	Country	Dependent/target	D	V	R	I	O	Method
Jovanis et al. (1991)	1982–1984	1800	USA	Accident frequency	✓	✓	✓			Log‐Linear Regression
Björnstig et al. (2005)	1994–2003	284	Sweden	Injury type	✓	✓			✓	Descriptive Statistics
Halpern et al. (2005)	2000	120	Israel	Injury type	✓				✓	Descriptive Statistics
Blower and Green (2010)	1999–2005	2252	USA	Accident involvement	✓	✓				Logistic Regression
Chimba et al. (2010)	2003–2007	4528	USA	Accident severity			✓		✓	Multinomial Logit
				Accident frequency						Negative Binomial
Barua and Tay (2010)	1998–2005	2662	Bangladesh	Accident severity		✓	✓		✓	Ordered Probit
Rahman et al. (2011)	2000–2007	9485	Canada	Accident severity	✓		✓			Logistic Regression
Kaplan and Prato (2012)	2005–2009	2576	USA	Accident severity	✓	✓	✓			Generalized Ordered Logit
Theofilatos et al. (2012)	2008	16,426^b^	Greece	Accident severity	✓	✓	✓		✓	Logistic Regression
Prato and Kaplan (2014)	2002–2011	3434	Denmark	Accident severity	✓	✓	✓			Generalized Ordered Logit
Feng et al. (2016)	2010–2016	1380	USA	Accident severity	✓	✓	✓			Ordered Logit
Yoon et al. (2017)	2010–2014	27,731	South Korea	Accident severity	✓	✓				Hierarchical Ordered
Sam et al. (2018)	2011–2015	33,694	Ghana	Accident severity	✓	✓	✓			Ordered Logit
Park et al. (2019)	2011–2015	9913	South Korea	Accident severity	✓	✓	✓	✓	✓	Hierarchical Linear Model
Tamakloe et al. (2020)	2010–2016	2997	South Korea	Accident severity	✓	✓	✓	✓	✓	Bivariate Copula‐based Model
Gu et al. (2020)	2012–2015	456	China	Accident frequency	✓	✓	✓	✓	✓	Spatiotemporal ZINB
Nasri and Aghabayk (2021)	2014–2016	5938	Iran	Accident severity	✓	✓	✓		✓	Logistic Regression
Shen et al. (2022)	2017–2019	14,678	UK	Accident severity	✓		✓		✓	Extend Hierarchical Ordered
L. Zheng et al. (2024)	2022	283	China	Injury severity	✓	✓	✓	✓	✓	PLS‐SEM
Zeng et al. (2025)	2009–2019	3404	China	Accident severity	✓	✓	✓		✓	Logistic Regression
Our study	2013–2018	15,076	China	Accident severity	✓	✓	✓	✓	✓	STM + XGBoost + SHAP

a D‐V‐R‐I‐O represent five categories of factors: Driver, Vehicle, Road, Institutional, and Other. (D) Driver: age, gender, license type, experience, violations, alcohol or drug use, fatigue, distraction, and risk tendency; (V) Vehicle: type, age, capacity, maintenance, braking and suspension systems, tire condition, and energy type; (R) Road: type, lanes, intersection density, pavement, visibility, weather, lighting, traffic control, and route complexity; (I) Institutional/organizational: operator and route details, safety policies, scheduling, training, and fleet management; (O) Other: day, week, month, accident text descriptions, pedestrian and cyclist exposure, passenger load, and injury details. b The dataset covers multiple vehicle types, but this study is included for its key insights into bus‐related accidents.

Prior bus accident studies typically follow two main approaches: one based on stakeholder‐reported information such as interviews and surveys, which provides insights into behavioral and organizational risk factors; and another based on the analysis of historical accident records, where statistical methods are employed to uncover patterns and quantify risk. Specifically, the first stream leverages qualitative or semi‐quantitative data to examine behavioral, perceptual, and organizational aspects of safety. For instance, Halpern et al. (2005) found that sudden acceleration or deceleration was the leading cause of noncollision injuries among Israeli bus passengers, underscoring the importance of improved driver training and passenger awareness. Similarly, although not focused exclusively on buses, Nævestad et al. (2015) and Warmerdam et al. (2017) examined broader traffic safety contexts, highlighting behavioral risks such as poor speed monitoring and limited seatbelt use, and emphasizing the importance of journey planning and communication in risk mitigation. More recently, L. Zheng et al. (2024) employed a hierarchical structures to incorporate latent‐variable structural modeling to reveal that driver behavior remains the most critical determinant of injury severity in bus accidents, while organizational safety management indirectly influences outcomes through its impact on driver conduct and vehicle maintenance practices.

The second, and more commonly adopted, stream of research relies on archival accident data to quantify and model risk factors through statistical methods. Early studies, such as those by Jovanis et al. (1991) and Björnstig et al. (2005), leveraged datasets to uncover recurring patterns in bus accident outcomes. Jovanis et al. (1991) identified rear‐end collisions as a primary source of injury in bus crashes within the Chicago metropolitan area, particularly affecting occupants of other vehicles. Similarly, Björnstig et al. (2005) reported a high incidence of whiplash injuries in Swedish urban bus crashes and advocated for preventive policy measures, including mandatory seatbelt use on long‐distance coaches.

Logistic Regression and its variants have been widely applied to examine the determinants of bus accident severity, particularly in capturing the probabilistic relationships between driver behavior, vehicle characteristics, and environmental conditions. These models are valued for their interpretability and ability to handle binary or ordinal outcomes. Blower and Green (2010) found that drivers with prior traffic violations and certain vehicle types were more likely to be involved in fatal crashes in the United States, emphasizing the critical role of experience and compliance in safety outcomes. Rahman et al. (2011) reported that adverse weather conditions in Canada increased the likelihood of accidents, though the resulting injuries were often less severe, suggesting that drivers may adopt more cautious behavior under poor visibility or slippery road conditions. Using national accident data from Greece, Theofilatos et al. (2012) showed that roadway geometry, lighting, and pavement quality significantly affect injury severity, with urban and rural areas exhibiting distinct risk profiles. In Iran, Nasri and Aghabayk (2021) demonstrated that higher speed limits, nighttime driving, and the presence of vulnerable road users were associated with greater fatality risk, underscoring the need for targeted infrastructure design and regulatory enforcement. More recently, Zeng et al. (2025) identified driver fatigue, road curvature, and vehicle age as persistent predictors of severe bus‐taxi collisions in China, further highlighting the contribution of both human and operational factors to accident outcomes.

Multinomial Logit, Ordered Logit, Generalized Ordered Logit, and Ordered Probit have also been widely used in the prior studies. Chimba et al. (2010) examined the frequency and severity of bus accidents in the United States through Negative Binomial and Multinomial Logit, finding that roadway design features (e.g., lane width, intersection density, and parking availability) significantly influence both. In Bangladesh, Barua and Tay (2010) used Ordered Probit and found that accident severity was higher during weekends and off‐peak hours, while the presence of enforcement measures such as police patrols and traffic‐calming infrastructure effectively mitigated risk. Extending this line of research, Gu et al. (2020) introduced a Spatiotemporal Random‐Effects Zero‐Inflated Negative Binomial (in short Spatiotemporal ZINB) model, revealing that higher passenger volumes and a greater proportion of male drivers on a route were associated with increased crash frequencies, thereby highlighting the influence of exposure and demographic composition on accident risk. Other studies have explored demographic, contextual, and hierarchical dimensions of accident severity. Kaplan and Prato (2012) employed Generalized Ordered Logit and identified that younger (< 25) and older (> 55) drivers faced a greater likelihood of fatal outcomes, particularly in high‐speed or intersection‐related crashes. Prato and Kaplan (2014) analyzed Danish accident data using a similar modeling framework and found that driver age, gender, and behavior‐along with road characteristics influenced crash severity. Feng et al. (2016) combined Clustering with Ordered Logit to show that road conditions, cyclist presence, and driver demographics influence crash severity differently across driver groups, highlighting substantial heterogeneity in risk behavior.

Hierarchical modeling has further advanced the understanding of accident severity by accounting for the nested structure of accident data and capturing the influence of factors operating at multiple levels. Using accident records from South Korea, Yoon et al. (2017) used Hierarchical Ordered Model to identify both micro‐level factors (e.g., speed, alignment, and weather conditions) and macro‐level influences (e.g., road infrastructure quality and emergency response capacity) as significant determinants of injury severity. Park et al. (2019) and Shen et al. (2022) extended the analysis to environmental conditions and bus‐pedestrian collisions, respectively, demonstrating how regional and operational contexts shape the distribution and severity of crash outcomes.

Collectively, these studies underscore the multifaceted and hierarchical nature of bus accident risk, shaped by the interaction among individual behavior, vehicle and roadway characteristics, organizational safety practices, and environmental conditions. However, prior research has relied predominantly on structured quantitative data, often overlooking the rich contextual and behavioral insights embedded in textual accident narratives. Furthermore, few studies have leveraged advanced machine learning techniques capable of modeling complex, nonlinear relationships while retaining interpretability. To address this gap, our study integrates structured and unstructured data within a novel machine learning framework that combines topic modeling, predictive modeling, and post hoc interpretation. Using a large‐scale, multi‐source dataset comprising over 15,000 bus accident records, the framework uncovers latent accident patterns, predicts severity outcomes, and interprets the underlying risk factors, providing a scalable and transparent foundation for evidence‐based transportation safety management.

## 3 Three‐Stage Machine Learning Analytical Framework

**FIGURE 1:**
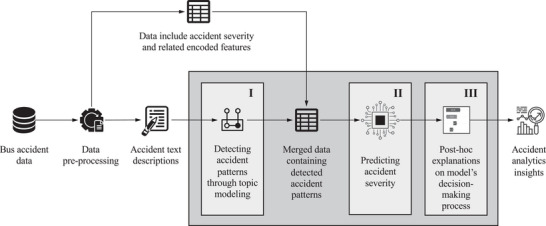
Schematic view of the proposed three‐stage analytical framework.

Figure 1 presents a schematic view of the proposed analytical framework, which comprises three stages: (i) accident pattern discovery, (ii) predictive modeling, and (iii) post hoc explanation. In Stage 1, latent semantic structures are extracted from bus accident narratives using a probabilistic topic model and transformed into semantically interpretable themes that capture contextual and severity‐related patterns; the resulting accident pattern proportions are then combined with other feature variables, along with the target variable of accident severity, to form a comprehensive dataset. Stage 2 focuses on predictive modeling of accident severity using the merged dataset. Stage 3 performs post hoc interpretability analysis of the fine‐tuned predictive model to quantify the contribution of each input feature to the predictions. The following subsections detail the methods used in each stage. The framework is modular, allowing alternative models to be integrated at any phase; the rationale and advantages of the chosen methods are discussed in depth.

### 3.1 Discovering Accident Patterns With STM

**FIGURE 2:**
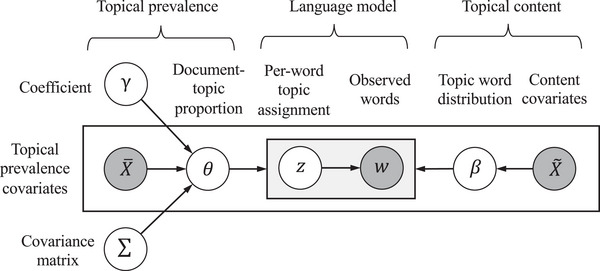
Schematic view of STM.

The first stage employs STM (Roberts et al. 2016) to identify bus accident patterns from textual descriptions of accidents. In this context, *patterns* (or *topics*) are conceptualized as distributions over a vocabulary of words, encapsulating semantically coherent “themes.” Assume a total of K patterns exist within the bus accident text descriptions. Each description is treated as a *document*, represented by d∈{1,…,D}, and each word within the description by $t\in \{1,\text{…},T_{d}\}$. The observed words $w_{d,t}$ are instances of unique terms from a vocabulary, indexed by v∈{1,…,V}. The STM is a generative graphical model, with its data‐generating process depicted in Figure 2. Each circle in the model represents a variable, where shaded circles denote observed variables and unshaded circles, hidden variables. The shaded rectangle symbolizes computational replications.

STM can be broadly divided into three components: (i) the topical prevalence model, (ii) the topical content model, and (iii) the core language model. The former controls how words can be allocated to bus accident patterns as a function of covariates. It allows the correlated topic proportion $\theta_{d}$, and the topical prevalence can be influenced by covariates ${\bar{X}}_{d}$ through a standard regression model, that is $\theta_{d}∼\mathrm{LogisticNormal}\left({\bar{X}}_{d}\gamma ,\Sigma \right)$, where γ is the coefficient vector and Σ is the covariance matrix. Covariates on the topical content $\tilde{X}$ allow for the rates of words used for each pattern to differ by covariate value. According to Roberts et al. (2014), the rate $\beta_{d,k,v}$ satisfies

(1) $$\begin{array}{cc} \beta_{d,k,v}∼ & \;\frac{\exp\{\zeta_{v}+\kappa_{k,v}+\kappa_{y_{d},v}+\kappa_{y_{d},k,v}\}}{\sum_{v}\exp\left\{\zeta_{v}+\kappa_{k,v}+\kappa_{y_{d},v}+\kappa_{y_{d},k,v}\right\}}, \end{array}$$

where $\zeta_{v}$ is the marginal log‐transformed rate of term v, and $\kappa_{k,v},\kappa_{y_{d},v},\kappa_{y_{d},k,v}$ are coefficients for the topical content model. Then, the core language model performs a two‐step generative process for each bus accident text description: (i) given the topic proportion $\theta_{d}$, for each word within a text description d, an accident pattern $z_{d,n}$ is sampled from a multinomial distribution $z_{d,n}∼\mathrm{Multinomial}\left(\theta_{d}\right)$; (ii) conditional on the pattern $z_{d,n}$, a word $w_{d,n}$ is sampled from a multinomial distribution $w_{d,n}∼\mathrm{Multinomial}\left(\beta_{d,z}\right)$.

In this stage, we employ a probabilistic topic modeling approach rather than neural or large language model (LLM)–based techniques such as BERT and GPT (Miao et al. 2016; Dieng et al. 2020; Srivastava and Sutton 2017; Devlin et al. 2019; Brown et al. 2020; Ouyang et al. 2022). Although neural and LLM‐based models can capture deep contextual semantics and dynamic word meanings, their advantages often come at the cost of interpretability, computational efficiency, and reproducibility. Kirilenko and Stepchenkova (2025) compared a range of topic modeling approaches in tourism research and found that, while neural and transformer‐based models perform well on short or noisy texts, probabilistic models such as Latent Dirichlet Allocation (LDA) (Blei et al. 2003) remain favored for their transparency and consistency. Building on these insights, our study adopts the STM, a state‐of‐the‐art probabilistic framework that extends LDA. STM provides a nuanced and contextualized understanding of semantic structures in bus accident narratives, capturing complex thematic patterns that conventional models assuming topic independence often overlook. Moreover, its Bayesian formulation enables uncertainty quantification in topic estimation, offering a transparent and interpretable foundation for data‐driven risk analysis and decision support. Despite its increasing adoption in tourism and business analytics (Tonidandel et al. 2022; B. Chen et al. 2025), STM has yet to be applied to risk management or transportation safety research.

### 3.2 Predicting Accident Severity With XGBoost

The accident patterns identified in the previous stage are combined with additional variables extracted from accident records, encompassing driver attributes, vehicle specifications, roadway and environmental conditions, and organizational characteristics, to form a comprehensive dataset for predictive modeling. The aim of this stage is to predict accident severity, which may be defined as a binary (e.g., severe vs. nonsevere) or multi‐class (e.g., minor, moderate, severe) outcome, consistent with prior studies reviewed in Section 2. In this study, severity is represented as a binary variable (see dataset description in Section 4.1), framing the task as a supervised two‐class classification problem. The proposed framework, however, remains adaptable to alternative formulations, including multi‐class or continuous severity representations, depending on the used dataset.

XGBoost (Chen and Guestrin 2016), a widely used predictive algorithm, is employed at this stage. It constructs an ensemble of decision trees in an additive manner, where each subsequent tree is trained to correct the residual errors of the preceding ones. This iterative process seeks to minimize a regularized loss function, defined as:

(2) $$\begin{array}{cc} L(f) & =\sum_{i}l\left(y_{i},\hat{y}i\right)+\sum m\Omega \left(\phi_{m}\right), \end{array}$$

where f denotes the overall prediction function, $l(y_{i},{\hat{y}}_{i})$ is a differentiable convex loss function that quantifies the discrepancy between observed and predicted outcomes, and $\Omega (\phi_{m})$ is a regularization term that penalizes model complexity to improve generalization. This formulation allows XGBoost to balance predictive accuracy with overfitting control. For readers seeking a more detailed explanation of the algorithm, please refer to Appendix 4 of the Supporting Information.

XGBoost is selected in this stage as the predictive model of choice due to its demonstrated performance and strong alignment with the characteristics of the task. Tree‐based methods, including decision trees and their ensemble extensions, have long been applied in risk analysis because of their ability to model complex decision‐making processes and accommodate heterogeneous data types. Early studies, such as Frohwein and Lambert (2000) and Frohwein et al. (2000), employed multiobjective decision trees to evaluate rare and severe event risks, establishing the foundation for structured and interpretable models in risk assessment. Similarly, Weng et al. (2013) developed a tree‐based logistic regression model for predicting work zone casualties, underscoring the practical value of decision‐tree frameworks in safety‐critical contexts. Building on these developments, recent advances in ensemble learning, particularly gradient boosting, have shown strong potential for transportation safety applications, with XGBoost achieving notable success in predicting accident severity and related risk outcomes (Khattak et al. 2024; Kabir et al. 2024; Q. Wang et al. 2025; Y. Li et al. 2025).

Beyond its empirical success, XGBoost offers several technical advantages that make it particularly well suited for large‐scale accident data. It supports efficient parallelized boosting, integrates regularization to mitigate overfitting, and robustly handles missing or sparse data. Compared with other advanced machine learning models that typically demand extensive data and intensive parameter tuning, XGBoost achieves high accuracy with relatively modest computational cost and fewer hyperparameter constraints. This balance between parsimony and performance reflects the principle of Occam's razor (Murphy 2022), whereby simpler models are preferred unless additional complexity yields substantial benefits. Empirical results presented in Section 4 also confirm XGBoost's strong predictive performance, reinforcing its suitability for this classification task. Nevertheless, the proposed framework remains intentionally modular, allowing for the substitution or integration of more advanced methods, such as deep neural networks or hybrid architectures, when future applications involve more complex data structures or demand greater representational capacity.

### 3.3 Explainable Analytics With SHAP

This stage provides post hoc explanations for the decisions made by the fine‐tuned XGBoost from the previous stage. The state‐of‐the‐art interpretability technique, SHAP (Lundberg and Lee 2017), is employed. Grounded in cooperative game theory, SHAP calculates Shapley values to quantify each feature's marginal contribution to an individual prediction. This enables a transparent understanding of how different features influence model outputs, both at the case level and across the entire dataset. Such interpretability is particularly critical in risk modeling, where understanding the rationale behind predictions supports accountability, trust, and the translation of analytical results into actionable safety insights.

Specifically, in cooperative game theory, groups of players (called *coalitions*) are the primary units of decision‐making and may enforce cooperative behavior. In supervised learning setting, features in an input data instance can be considered as players in a coalition and the Shapley value for one feature is defined as the average marginal contribution of the a feature value across all possible coalitions. Let $\xi_{j}\left(f,x\right)$ denote the Shapley value representing the impact of feature value j on the prediction of the XGBoost classifier f for the given input data x. We can compute it using a weighted sum that represents the impact of each feature being added to the model averaged over all possible orders of features being introduced. That is,

$$\begin{array}{cc} \xi_{j}\left(f,x\right)= & \sum_{W\subseteq \{1,\text{…},J\}\setminus \{j\}}\frac{|W|!(J-|W|-1)!}{J!}\left[f_{x}\left(W\cup \left\{j\right\}\right)-f_{x}\left(W\right)\right], \end{array}$$

where J is the size the feature vector, and W is the subsect of {1,…,J}∖{j}.

SHAP enjoys several desirable theoretical properties that contribute to its robustness and credibility as a model‐agnostic explanation technique. Specifically, it satisfies three key axioms derived from cooperative game theory: (i) *local accuracy* (also known as *efficiency*), (ii) *missingness*, and (iii) *consistency*. These properties ensure that SHAP values provide meaningful and mathematically grounded attributions of feature importance. For a detailed explanation of SHAP axioms, please refer to Appendix 6 of the Supporting Information.

These properties set SHAP apart from many other interpretability methods, making it particularly well‐suited to risk modeling applications where transparency, fairness, and theoretical rigor are crucial  (Khattak et al. 2024; Q. Wang et al. 2025). One of SHAP's main strengths lies in its model‐agnostic design, allowing it to be applied consistently across diverse machine learning models. This flexibility is especially beneficial in analytical frameworks where different predictive models may be evaluated or combined. In addition, SHAP supports both instance‐level and global interpretability, offering insight into individual predictions while also summarizing overall feature importance across the dataset. This dual capability distinguishes SHAP from alternative approaches such as Local Interpretable Model‐Agnostic Explanations (LIME) (Ribeiro et al. 2016) and Confident Itemsets Explanation (CIE) (Kenny et al. 2021), reinforcing its value for interpretable and accountable risk analysis.

## 4 Experiments

This section presents our experiments, covering the dataset, key experimental steps, and result analysis. The evaluation assesses the predictive accuracy and interpretability of the proposed framework, and demonstrates its practical applicability to real‐world accident severity prediction.

### 4.1 Dataset and Preprocessing

The bus accident dataset used in this study is obtained through a research collaboration with public transport operators in a Tier‐2 city in Jiangsu Province, China. It contains 15,076 bus accident records across 243 routes operated by 36 companies between 2013 and 2018. To comply with non‐disclosure agreements and institutional ethical standards, all sensitive commercial and personal information has been anonymized or replaced with pseudonymous identifiers in publication. This includes the names of bus companies, route identifiers, driver information, and the identities of injured individuals.

The target variable, accident severity, was encoded by the data provider as a binary indicator (severe vs. nonsevere) based on insurance claim records and repair cost information. The dataset contains 17 feature variables across the five feature categories summarized in Table 1, including accident timestamp, unstructured description, cause, type, weather conditions, company and route identifiers, vehicle specifications (length, energy type, brake and suspension systems), and driver attributes (ID, gender, age, education, and driving experience).

The original dataset was in Chinese, including variable names, non‐numerical categorical values, and unstructured accident narratives. To ensure consistency in analysis and facilitate publication, all textual elements were translated into English. Variable names and categorical values (e.g., accident type, weather condition, energy type) were translated manually to preserve semantic precision. Automotive‐related terms and technical jargon were standardized during translation. For example, the category 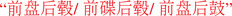 describing the bus brake system was translated as “Front disc and rear drum brake.” Details of these translations are provided in Appendix 1 of the Supporting Information.

For the large corpus of unstructured accident descriptions, the Google Cloud Translation API is employed, leveraging pretrained neural machine translation models to achieve consistent and context‐aware translation quality (Lucas et al. 2015).^1^ The translated texts were then manually reviewed to correct contextual nuances and resolve inconsistencies. Because our data were obtained through a research collaboration with public transport operators and the accident narratives were written for police documentation and insurance claim purposes, they are generally informative, concise, and well‐structured. Consequently, the translation quality is high and retains the essential contextual and semantic content of each record. The corpus contains very little jargon or technical terminology. It should be noted, however, that the names of roads, streets, and buildings are rendered in pinyin, as are the names of bus drivers or injured individuals. In the publication document, company and personal names are anonymized to comply with ethical requirements. For reference, representative accident descriptions in both Chinese and English are presented in Appendix 2 of the Supporting Information.

### 4.2 Latent Accident Patterns

Prior to training the STM, comprehensive text preprocessing was conducted on the translated English accident narratives. The procedure followed the standard workflow implemented in the stm package in R (Roberts et al. 2019), including lowercasing, tokenization, removal of punctuation and numerical values, and elimination of stop words. The package employs an approximate variational Expectation‐Maximization (EM) algorithm for model estimation but does not compute topic exclusivity when covariates on topic content are specified. In our implementation, bus length, brake system, driver gender, driver education, weather condition, month, and hour were included as covariates on topic prevalence.

**FIGURE 3:**
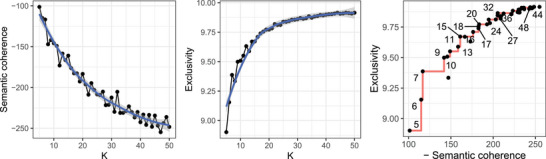
Selection of the optimal number of patterns for the STM.

The number of latent patterns (K) is a key hyperparameter in topic modeling that must be specified prior to training. Following established fine‐tuning procedures (Roberts et al. 2014; Hu et al. 2019; B. Chen et al. 2025), we systematically searched K values ranging from 5 to 50 and evaluated alternative models using two complementary metrics‐semantic coherence (Mimno and McCallum 2008) and exclusivity (Airoldi and Bischof 2016)‐to guide model selection (see Appendix 2 of the Supporting Information for metric definitions). Figure 3 presents the STM model selection results. As expected, semantic coherence decreases while exclusivity increases as K grows. The third subplot illustrates the trade‐off between these two metrics, with the red stepwise line marking the Pareto frontier that represents the optimal balance between exclusivity and coherence. Among the candidate models, those with K=7,15,20, and 32 exhibited the most favorable trade‐offs. After qualitative comparison of the generated topic structures and their representative accident descriptions (see Appendix 3 of the Supporting Information), the model with K=15 was found to produce the most semantically coherent and interpretable patterns. Therefore, K=15 was adopted for subsequent analyses.

Table 2 summarizes the 15 latent patterns extracted from the textual descriptions of bus accident records. The “Top words” column lists the most probable terms inferred from the topic‐word distribution. Because each accident narrative typically represents a mixture of several corpus‐wide themes, we manually reviewed representative descriptions with high topic probabilities to facilitate interpretation (see Appendix 2 of the Supporting Information). This manual inspection, together with the examination of top‐ranked words, enabled us to assign concise and meaningful descriptive labels to each topic. The resulting human‐interpreted summaries, reported in the “Summary” column, provide a clear overview of the dominant semantic patterns uncovered by the STM.

**TABLE 2:** Summary of identified patterns for text description of bus accident records.

Pattern	Summary	Top words ^a^	Proportion
1	Collision arises from bus reversing (with damage description)	mirror, station, glass, reversing, broken, windshield, right	5.94%
2	Rear‐end collision between vehicles (at a detailed place)	road, driving, west, east, intersection, Tongjiang, Jinling	7.54%
3	Sideswipe collision between vehicles (due to changing lanes)	car, right, left, front, north, south, direction	18.92%
4	Rear‐end collision (waiting for the green light, by cyclist)	electric, rear‐ended, bike, tricycle, light, cyclist, motorcycle	4.16%
5	People injury accidents (with a detailed injury description, people sent to hospital)	hospital, female, south, old, north, injured, years	4.32%
6	Sideswipe collision between vehicles (no detailed place and no damage description)	car, sideswipe, drove, party, going, walking, fled	8.73%
7	Sideswipe collision between vehicles (no detailed place but with direction and damage description)	damaged, bus, east, west, left, sideswipe, rear	9.21%
8	Sideswipe collision between vehicles (at a detailed place, with direction but no damage description)	north, south, truck, traveling, going, garden, middle	5.04%
9	Sideswipe collision between vehicles (left hand turns)	causing, rear‐end, turning, cars, taxi, turn, two	2.24%
10	Sideswipe collision with a fixed object (no detailed place and no damage description)	time, place, arrived, went, Zhang, Chen, Wang	14.87%
11	Sideswipe collision between vehicles (at a detailed place, no direction and damage description)	collided, section, bridge, descended, street, village, mentioned	3.60%
12	Sideswipe collision with a fixed object (at a detailed place, with damage description)	hit, guardrail, park, wipe, roadside, entering, platform	2.11%
13	No collision but people injuries with detailed description	passenger, injured, fell, sudden, stop, passengers, door	7.86%
14	Sideswipe or rear‐end collision (mainly due to road issues)	vehicle, front, vehicles, collision, due, slipped, venue	2.13%
15	Sideswipe or rear‐end collision (no detailed place and no damage description)	car, point, sideswiped, left, body, small, drive	3.32%

a Words within each topic with the highest probability inferred from the topic‐word distribution parameter.

Specifically, Pattern 1 is about collisions arising from bus reversing in stations or car parks, with windshield or mirror broken. Pattern 2 is about to rear‐end collisions (mainly the bus rear‐ends a car, truck, taxi and so on) at a specific location such as Tongjiang road and Jinling Road. The name of both roads also appears in the column “Top words.” Pattern 3 describes sideswipe collisions due to a car in the same direction changing lanes. In mainland China, all traffic should drive on the right‐hand side of the road. Therefore, slower lanes and slip roads are on the right, and middle and left lanes should only be used to overtake slower traffic. There are two scenarios in this pattern. First, a car travels fast and changes lanes from left lane to right lane so the left door or front corner of the bus gets damaged. Second, a car accelerates and changes lanes from the right lane to the left lane so the right door or front corner of the bus gets damaged. Pattern 4 is about collisions that the bus is rear‐ended by electric scooters, motorcycles, or tricycles when it is waiting at the traffic light. Pattern 5 describes bus accidents with injured passengers or pedestrians. The description contains a lot of details, including the bus movement direction, the name and age of injured people, and the specific damage to the body. Many records show severe injuries (e.g., bone fractures, head hematoma) so injured people have been taken to hospital. Pattern 6 is about sideswipe collisions between vehicles. There is no detailed location and no damage description. Pattern 7 describes sideswipe or rear‐end collisions between vehicles. Many records do not contain detailed accident locations but they mention that vehicles travel on a road which is along the east‐west direction. Also, detailed damages of the bus are described such as bumper, mirror, and taillights. Pattern 8 is also about sideswiping or rear‐end collisions between vehicles. Different to Pattern 7, many record descriptions provide a detailed accident location, and vehicles travel on a road which is along the north‐south direction. However, detailed damages to the bus are not provided. Pattern 9 describes sideswipe or rear‐end collisions with cars or pedestrians when the bus turns left or right at the intersection. Pattern 10 is about sideswipe collisions with a fixed object. There is no detailed accident location or damage description. However, in many text descriptions, the bus driver's surname is provided (e.g., Zhang, Chen, Wang). Pattern 11 is about sideswipe collisions between vehicles. Different from Patterns 6–8, many text descriptions contain a detailed location but no bus movement direction and damage details. Pattern 12 describes sideswipe collisions with a fixed object. Different from Pattern 10, the accident location and damage details (e.g., water tank, rear taillights) are provided in many record text descriptions. Pattern 13 is about bus sudden stop accidents causing passenger injuries. Some descriptions provide the accident location. However, compared to Pattern 5, many text descriptions are brief, do not provide injury details, and the injured people are not taken to the hospital. Pattern 14 describes sideswipe or rear‐end collisions mainly due to road issues such as road construction, small turning angles, and so on. Pattern 15 is about sideswipe or rear‐end collisions with a car. Compared to other patterns, many text descriptions are very brief, do not contain detailed accident location and damage details.

**FIGURE 4:**
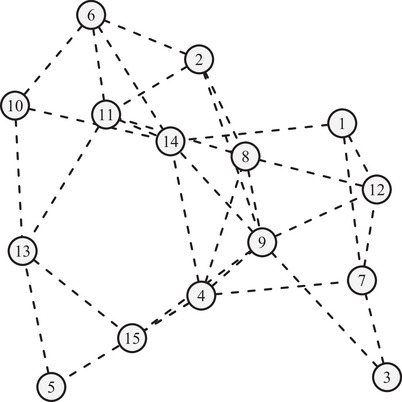
Network graph of the identified patterns from STM.

The identified patterns provide more details about in which scenario these bus accidents occur. They are consistent with much existing literature in traffic accident analysis and prevention. For example, in Pattern 5, we find that many injured people are elderly females. This is consistent with the findings of Wilmut and Purcell (2021) that elderly people are more likely to be seriously injured in road traffic accidents. To better illustrate the relationship among these patterns, we use the Meinshausen‐Buhlmann method (Zhao et al. 2012) to visualize the network structure. As depicted in Figure 4, each node of the graph represents a pattern, and two patterns are connected by a link (or edge) if they tend to co‐occur with a high probability. It reveals the correlation of patterns, which further verifies our pattern labeling. For example, Patterns 2, 6, 8, 10, 11, and 14 are about sideswipe or rear‐end collisions but with fewer damage details; Patterns 1, 3, 7, and 12 provide damage details for different sideswipe or rear‐end collisions; Patterns 5 and 13 describe bus accidents with people injuries.

### 4.3 Severity Prediction and Post Hoc Explanations

**FIGURE 5:**
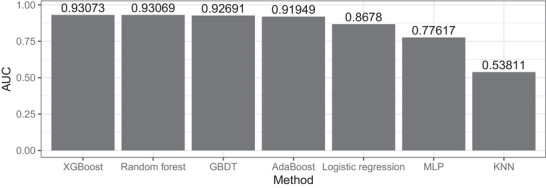
Cross‐validation performance of machine learning algorithms based on AUC.

XGBoost was implemented to predict bus accident severity using its open‐source Python library in a single‐machine environment running Python 3.^2^ Categorical variables were transformed into binary indicators via one‐hot encoding, and model fine‐tuning was conducted using 10‐fold cross‐validation combined with a grid‐based hyperparameter search. This procedure was applied not only to XGBoost but also to several commonly used machine learning algorithms for benchmarking, including Random Forest, Gradient Boosting Decision Trees (GBDT), AdaBoost, Multi‐Layer Perceptron (MLP), K‐Nearest Neighbors (KNN), and Logistic Regression. While these methods have been used in some previous traffic accident studies (see Section 2), their application to bus accident analytics remains limited. The specific hyperparameter configurations used during model tuning are provided in Appendix 5 of the Supporting Information. Model performance was evaluated using the area under the receiver operating characteristic curve (AUC), a standard metric for assessing the discriminative ability of binary classifiers. As shown in Figure 5, XGBoost consistently achieved the highest AUC scores across multiple folds, outperforming all benchmark models. This result supports the use of XGBoost as the primary predictive model within the proposed framework. Furthermore, the framework is inherently modular and can readily accommodate alternative or more advanced machine learning algorithms if required by future datasets or analytical objectives.

**FIGURE 6:**
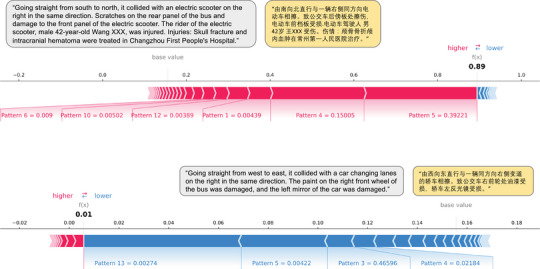
Empirical examples of local explanations on the contributions of features towards the bus accident severity.

The SHAP framework was implemented in Python 3 via the TreeExplainer (Lundberg et al. 2020) to provide post hoc interpretation of the predictions produced by the fine‐tuned XGBoost model. Figure 6 presents two empirical examples of local explanations, illustrating the contribution of different features to the predicted severity of bus accidents. In these examples, the original Chinese accident descriptions are highlighted in yellow and their English translations in gray. The colors red and blue denote positive and negative contributions to the prediction, respectively. The first (top) example corresponds to a severe bus accident, where f(x)=0.89 is substantially higher than the base value E[f(x)]=0.1556. High values of Patterns 4 and 5, combined with low values of Patterns 1, 6, 10, and 12, increase the SHAP value, reinforcing the “severe” classification. The second (bottom) example, classified as nonsevere with f(x)=0.01, shows the opposite effect: low values of Patterns 4, 5, and 13, together with a high value of Pattern 3, reduce the SHAP value below the base expectation.

**FIGURE 7:**
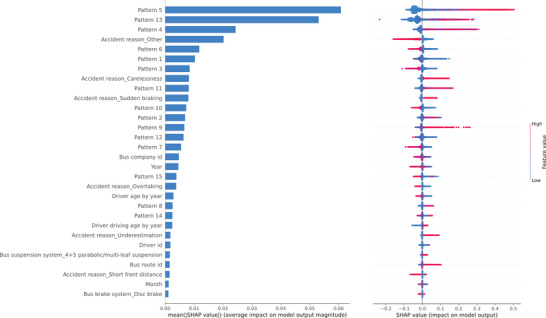
Plots of (left) global feature importance and (right) local explanation summary.

While these local explanations reveal detailed reasoning behind each classification, their insights are case‐specific. To obtain a broader perspective, we aggregated many local explanations to examine global feature importance. Features with large absolute Shapley values exert stronger influence on model predictions; thus, averaging these absolute values across all data instances provides a measure of overall feature impact. Figure 7 displays the top 30 globally important features along with a summary of local explanation distributions. In the summary plot, red dots represent high feature values and blue dots represent low ones, while the *x*‐axis denotes the SHAP contribution for each feature across samples. Notably, several latent patterns identified in the first stage appear among the most influential predictors, confirming that incorporating STM‐derived features from unstructured text substantially enhances both model performance and interpretability.

Figure 7 highlights Patterns 5, 13, and 4 as the most influential factors associated with severe bus accidents. As discussed in Section 3.1, these patterns capture accident scenarios involving passenger or pedestrian injuries and therefore contribute positively to severity classification. Pattern 5 corresponds to incidents involving injured passengers or pedestrians (often older females), aligning with findings reported by Björnstig et al. (2005), Prato and Kaplan (2014), Sam et al. (2018), and Wilmut and Purcell (2021). Pattern 13 primarily reflects noncollision injuries due to sudden braking or acceleration, with top words such as “fell”, consistent with Halpern et al. (2005), who identified sudden deceleration as a major mechanism of injury among standing passengers. Pattern 4 captures situations where buses are rear‐ended by electric scooters, motorcycles, or tricycles while waiting at traffic lights, resulting in injuries to the riders‐typically due to their inattention or misjudgment.

Left‐hand turns (Pattern 9) also emerge as a key feature contributing to severe bus accidents. In China, where vehicles are left‐hand drive and travel on the right‐hand side, left turns (or U‐turns) at intersections require wider turning angles and greater attentional demands. Drivers must simultaneously monitor cross‐traffic, signals, and pedestrians, which increases cognitive load and risk. For instance, if a bus driver follows a car through a left turn and assumes it will complete the maneuver, failure to check its progress may lead to rear‐end collisions or abrupt stops causing passenger injuries. Furthermore, blind spots during turning maneuvers can endanger pedestrians (Park et al. 2019; Yoshitake and Shino 2018). According to the National Highway Transportation Safety Administration (2010), left‐hand turns account for approximately 22% of all vehicle crashes in the United States, often resulting from obstructed views, misjudged gaps or speeds, inadequate surveillance, or false assumptions about other drivers' actions.

Rear‐end and sideswipe collisions exhibit mixed effects on accident severity. Positive contributions are observed for patterns containing detailed spatial or contextual information (e.g., Patterns 2, 8, 9, and 11), whereas brief or generic descriptions (e.g., Patterns 3, 6, 7, and 15) tend to reduce SHAP values. Rear‐end collisions occurring during reversing maneuvers in depots or car parks (Pattern 1) and sideswipes involving fixed objects (Patterns 10 and 12) are typically associated with minor property damage rather than serious injury. Additionally, results suggest that newly established bus companies or routes tend to exhibit better safety performance. This may be attributed to (i) more recent adoption of formal safety and risk management systems and (ii) greater accountability and vigilance among newer employees.

## 5 Discussion

This study offers both methodological and empirical contributions to the field of risk analysis and transportation safety. Methodologically, it presents a unified three‐stage machine learning framework that integrates structured data (e.g., driver demographics, vehicle specifications, road conditions) with unstructured accident narratives to support a richer, more contextualized analysis of accident risk. The framework combines topic modeling, non‐linear severity prediction, and model‐agnostic post hoc interpretation to balance predictive accuracy with explainability and computational efficiency. For explainability, the STM in Stage I extracts interpretable themes from accident descriptions, while SHAP in Stage III enables interpretation of model outputs—facilitating actionable insights for drivers, operators, and policymakers alike. In terms of efficiency, the selected models in stages (i.e., STM, XGBoost, and SHAP) are lightweight, scalable, and capable of running on standard hardware, eliminating the need for specialized computational infrastructure. This makes the framework particularly well‐suited for large‐scale, real‐time, or resource‐constrained deployments in operational risk environments.

Another notable methodological advantage of the proposed framework is its modular and extensible design, which allows for easy substitution and adaptation of components to meet different analytical needs. In Stage I, STM is used to extract interpretable topics from accident text narratives, offering an effective compromise between thematic coherence and computational efficiency. This stage, however, can be enhanced by more advanced text modeling approaches, such as neural topic models grounded in variational inference and semantic embeddings or LLMs that are capable of capturing deep contextual meaning from narrative data. Stage II applies XGBoost for severity prediction but it can also be replaced with tailored deep learning architectures when greater predictive capacity is required. Stage III leverages SHAP for post hoc explanation, providing a robust, model‐agnostic approach to interpreting variable importance. While SHAP is considered a state‐of‐the‐art method, alternative techniques, such as LIME, or CIE, can also be integrated, depending on application‐specific requirements and stakeholder needs.

Building on this methodological foundation, the analysis yields several actionable insights for accident prevention. At the individual level, patterns extracted from narrative data reveal that carelessness, sudden braking, and misjudgment are dominant causes of severe injury incidents, while overtaking and short following distances are more associated with minor collisions. These findings highlight the need for improved route awareness and schedule adherence to reduce abrupt maneuvers. The integration of Advanced Driver Assistance Systems could further enhance safety by supporting decision‐making related to acceleration, braking, and steering (Yan et al. 2007). Although urban buses in many countries are not required to be equipped with seatbelts, drivers should ensure passengers are securely positioned before departure. Routine announcements, such as “Please hold on, the bus is about to move,” play an important role, particularly for older or vulnerable passengers‐and could be expanded to context‐specific warnings in high‐risk zones.

At the organizational level, the analysis highlights a particularly vulnerable subgroup: young but experienced drivers. While their tenure suggests familiarity with operations, this group may be prone to behavioral risk factors such as overconfidence, impulsivity, and sensation seeking (Clarke et al. 2006; Rahman et al. 2011), increasing their likelihood of involvement in severe incidents. By contrast, newly qualified drivers, often closely monitored and recently trained, tend to exercise greater caution behind the wheel. These findings point to the need for more nuanced interventions that go beyond experience alone. Targeted initiatives such as risk‐awareness workshops and performance‐based safety incentive schemes can help address this gap (Carrera et al. 2020; Keller et al. 2021). Complementing these efforts, in‐vehicle monitoring technologies, such as telematics systems or mobile applications, can offer continuous feedback on driving behavior and reinforce safe practices. Metrics like eco‐driving scores have demonstrated success in promoting both road safety and fuel efficiency (Baecke and Bocca 2017).

From a policy perspective, our findings point to two key strategies. First, public education campaigns targeting passengers, pedestrians, and cyclists can raise awareness of safe behaviors and reduce accident vulnerability. For example, passengers are safer when facing forward and holding handrails, as sideways standing increases the risk of head or chest injuries in the event of a sudden stop (Yao et al. 2021). Cyclists and pedestrians should be especially cautious near intersections, where visibility and turning dynamics introduce heightened risks. Awareness campaigns should also reflect regional traffic patterns, including the risks associated with left‐ or right‐hand turns, depending on the country's driving orientation. Second, infrastructure improvements, such as installing dedicated turning lanes at high‐risk intersections, can help eliminate conflict points and enhance road safety (Department of Transportation Federal Highway Administration 2009).

## 6 Conclusion

We have proposed a novel explainable machine learning framework for bus accident analytics that integrates three components: topic modeling, tree‐based ensemble learning, and model‐agnostic post hoc interpretation. Using a comprehensive dataset from a Tier‐2 city in China, the framework uncovers latent patterns in accident narratives and produces interpretable predictions of accident severity. The empirical results provide actionable insights for accident prevention and demonstrate the framework's value for policymakers and practitioners in the transportation sector. Methodologically, the framework advances explainable analytics in risk analysis and transportation safety by integrating structured and unstructured data within a modular architecture that balances predictive accuracy, interpretability, and computational efficiency. Its flexible design also allows adaptation to domains beyond transportation and risk management. The successful application to bus accident data underscores its contribution as both a robust research framework and a practical decision‐support tool.

This paper paves the way for several promising avenues of future research. As the primary objective of this study is to develop and demonstrate the proposed methodological framework, a formal expert evaluation was not included within the current scope. In future research, such an evaluation could be incorporated to further validate and extend the framework through expert assessment and practical feedback. Future extensions of the framework could also integrate image or video data to provide pixel‐level contextual information and a more nuanced understanding of accident scenarios. Incorporating such visual data would enable scalable, automated analysis through advances in computer vision, thereby enriching the interpretation of accident dynamics. This direction holds significant potential for advancing safety research and represents a promising avenue for future investigation. More broadly, this work aims to encourage continued exploration of big data and machine learning approaches in business and risk analytics, fostering innovation in evidence‐informed decision‐making.

## Supporting information

Supporting Table 1 Mapping of Chinese categorical values to English translations for automotive‐related terms and jargon.
Supporting Table 2 Representative examples of bus accident descriptions generated from the STM.
Supporting Table 3 Hyperparameter tuning of the XGBoost and benchmarked algorithms.

## Endnotes

1 URL: https://console.cloud.google.com/apis/api/translate.googleapis.com

2 URL: https://xgboost.readthedocs.io
