# Supplementary material for: From Prediction to Prevention: Using Text Mining and Explainable Machine Learning for Urban Bus Accident Analytics
Source: Risk Anal. 2026 Jan 30;46(1):e70183. doi: 10.1111/risa.70183 (PMC12857609; doi:10.1111/risa.70183)
Supplement: Supplementary file 1 — Supporting Table 1 Mapping of Chinese categorical values to English translations for automotive‐related terms and jargon. Supporting Table 2 Representative examples of bus accident descriptions generated from the STM. Supporting Table 3 Hyperparameter tuning of the XGBoost and benchmarked algorithms. [file RISA-46-0-s001.pdf]

Supporting Information

From Prediction to Prevention: Using Text Mining and Explainable Machine Learning for Urban Bus Accident Analytics

1 | AUTOMOTIVE-RELATED TERMS AND JARGON

The original dataset used for this research was in Chinese, including variable names, non-numerical categorical values, and unstructured accident narratives. To ensure consistency in analysis and facilitate publication, all textual elements were translated into English, with variable names and categorical values translated manually to preserve semantic precision. The mapping of translations for automotive-related terms and technical jargon is provided in Table 1 for reference.

TABLE 1 Mapping of Chinese categorical values to English translations for automotive-related terms and jargon.

Variable	Category value (in Chinese)	Category value (translation in English)
Bus energy type	柴油	Diesel
	天然气	Natural gas
	电	Electric
Bus brake system	前盘后毂/ 前碟后毂/ 前盘后鼓	Front disc and rear drum brake
	盘式	Disc brake
	毂式/ 鼓式	Drum brake
Bus suspension system	前3后4	3+4 parabolic/multi-leaf suspension
	前3后5	3+5 parabolic/multi-leaf suspension
	前4后4	4+4 parabolic/multi-leaf suspension
	前4后5	4+5 parabolic/multi-leaf suspension
	前8后8	8+8 parabolic/multi-leaf suspension
	前8后9	8+9 parabolic/multi-leaf suspension
	前9后9	9+9 parabolic/multi-leaf suspension
	气囊	Air suspension

2 | TOPIC MODEL EVALUATION METRICS

The number of patterns (or topics, in topic modeling terminology) K is a hyperparameter that needs to be explicitly specified to control the model training process. Two evaluation metrics are used in our model selection: semantic coherence and exclusivity.

The *semantic coherence* metric measures how well the words within a topic co-occur in the same documents. As defined by Mimno *et al.* (2011), it estimates the conditional likelihood of word co-occurrences, capturing how frequently the top words of a topic appear together within accident narratives. Given a list of the Q most probable words in pattern k, the semantic coherence score C_k is computed as follows:

$$C_k = \sum_{e=2}^{Q} \sum_{\epsilon=1}^{e-1} \log \left\{ \frac{\Theta(v_e, v_\epsilon) + 1}{\Theta(v_\epsilon)} \right\}, \tag{1}$$

where $\Theta(v_e, v_\epsilon)$ represents the number of times that words v_e and v_ϵ appear together in the accident record text.

On the other hand, *exclusivity* measures how unique the top words are to a particular topic. It reflects the extent to which a topic's top words are unlikely to appear among the top words of other topics and can be quantified using the frequency–exclusivity (FREX) score (Airoldi and Bischof, 2016), defined as follows:

$$\text{FREX}_{k,v} = \left[\frac{\pi}{\text{ECDF}(\beta_{k,v}) / \sum_{\tilde{k}=1}^{k} \beta_{\tilde{k},v}} + \frac{1-\pi}{\text{ECDF}(\beta_{k,v})} \right]^{-1}, \tag{2}$$

where ECDF is the empirical cumulative distribution function and π is the weight. In our used R package `stm`, $\pi = 0.7$ by default (Roberts *et al.*, 2019).

In general, a model with higher coherence produces more semantically meaningful topics, whereas higher exclusivity indicates that topics are well-separated and less redundant. Because the two metrics often trade off against each other, the optimal K is typically chosen by balancing interpretability (coherence) and differentiation (exclusivity). These metrics jointly help assess the interpretability and distinctiveness of the learned patterns.

3 | BUS ACCIDENT DESCRIPTION EXAMPLES

The Google Cloud Translation API was used to translate the large corpus of unstructured bus accident descriptions originally written in Chinese.[1] This service employs pre-trained neural machine translation models capable of generating context-aware and linguistically consistent translations. Table 2 presents representative examples of both the original Chinese accident narratives and their English translations, corresponding to the accident patterns (topics) identified through the Structural Topic Model

[1] URL: https://console.cloud.google.com/apis/api/translate.googleapis.com

(STM) (Roberts *et al.*, 2019). To ensure confidentiality, all privacy-sensitive and commercially sensitive information related to bus companies and individuals has been anonymized and masked in black.

TABLE 2 Representative examples of bus accident descriptions generated from the STM.

Pattern	Accident description (in Chinese)	Accident description (translation in English)
1	[redacted]公司立体停车场三楼倒车时撞立柱，后挡风破。	When reversing on the third floor of the three-dimensional parking lot of the [redacted] Company, the bus hit a column and the rear windshield was broken.
1	[redacted]公司停车场二楼倒车时后挡风撞立柱	When reversing on the second floor of the parking lot of the [redacted] Company, the rear windshield hits the column.
1	[redacted]公司车间通车时，右车厢第二块玻璃撞	When moving in the workshop of the [redacted] Company was opened to traffic, the second glass of the right carriage was broken.
1	火车站站回车场倒车时撞破后挡风	The rear windshield was broken when reversing at the train station back to the parking lot.
1	[redacted]公司车间倒车撞立柱，后挡风破	The workshop of the [redacted] Company reversed and hit the column, and the rear windshield was broken.
2	飞龙路长江路由东向西行驶追尾面包车	Rear-ending van driving east to west on Feilong Road and Yangtze River Road.
2	黄河西路包家塘，由东向西行驶追尾一辆卡车	Baojiatang on West Yellow River Road, driving from east to west, rear-ending a truck.
2	飞龙东路永宁花园由东向西行驶追尾宝马	Driving from east to west in Yongning Garden on Feilong East Road and rear-ending a BMW.
2	飞龙路家乐福，由西向东行驶追尾一辆出租车	Carrefour on Feilong Road, driving from west to east, rear-ending a taxi.
2	龙城大道巫山路由东向西行驶追尾一辆宝马X3	Longcheng Avenue Wushan Road, driving east to west, rear-end a BMW X3.
3	由北向南与同方向右侧变道轿车相擦。致己车右前角受损，对方车左侧受损。	From north to south, it collided with a car that changed lanes on the right in the same direction. Damage to the right front corner of the own car and damage to the left side of the opponent's car.
3	由北向南与同方向右侧变道轿车相擦。致己车右前角受损，对方车左侧受损。	From north to south, it collided with a car that changed lanes on the right in the same direction. Damage to the right front corner of the own car and damage to the left side of the opponent's car.
3	由北向南与同方向右侧变道轿车相擦。致己车前门处受损，对方车左前侧受损。	From north to south, it collided with a car that changed lanes on the right in the same direction. Damage to the front door of your own car and damage to the left front side of the opponent's car.
3	由北向南与同方向右侧变道轿车相擦。致己车右前角受损，对方车左后侧受损。	From north to south, it collided with a car that changed lanes on the right in the same direction. Damage to the right front corner of your own car and damage to the left rear of the opponent's car.
3	由南向北与右侧同方向变道轿车相擦。致己车右前角受损，对方车左侧受损。	From south to north, it sideswipe a car that changed lanes in the same direction on the right. Damage to the right front corner of the own car and damage to the left side of the opponent's car.
4	叶家码头由西向东行驶被一电动三轮车追尾。	Yejia Wharf was running from west to east and was rear-ended by an electric tricycle.
4	市民广场等红灯时被追尾	Being rear-ended while waiting for a green light at the Civic Square.
4	由西向东被由南向北电动车追尾报警后电动车驾驶员自行离开	After being rear-ended by an electric bike from south to north from west to east, the driver of the electric bike left on his own after calling the police.

4	由西向东等信号灯时被后方电动三轮车追尾。致己车右后尾灯受损。	He was rear-ended by an electric tricycle behind while waiting for the signal light from west to east. Damaged the right rear tail light of the vehicle.
4	行驶至此，通过路口时与一闯红灯电动车擦致使骑车人受伤。	At this point, the cyclist got injured by sideswipe an electric bike that ran a red light when passing through the intersection.
5	由北向东与由北向南的行人相撞。致行人倒地受伤。[redacted]，男，83岁，常州中医院12楼住院。肋骨骨折、头部血肿。电话：[redacted].	From north to east, it collided with pedestrians from north to south. Pedestrians fell to the ground and got injured. [redacted], male, 83 years old, was hospitalized on the 12th floor of Changzhou Hospital of Traditional Chinese Medicine. Rib fractures, head hematoma. Tel: [redacted].
5	由西向东遇情况制动时致车内两名乘客受伤。[redacted]，男，45岁。经检查无碍。[redacted]，女，41岁。右肩锁骨关节半脱位可能。常州一院就医。未住院。	Two passengers in the car were injured when braking from west to east. [redacted], male, 45 years old. Checked fine. [redacted], female, 41 years old. Right acromioclavicular joint subluxation possible. Changzhou First Hospital for medical treatment. Not hospitalized.
5	由北向南因制动致车内客伤。[redacted]，男，69岁。面部撞伤。武进中医院就医，软组织挫伤。	Passengers in the car were injured due to braking from north to south. [redacted], male, 69 years old. Facial bruise. Wujin Traditional Chinese Medicine Hospital for treatment, soft tissue contusion.
5	由南向北因避让由西向东的轿车，制动时致车内一老年女性乘客腰部摔伤。[redacted]，女，67岁。T12腰椎压缩性骨折。常州一院就医。电话：[redacted]	From south to north, an elderly female passenger in the car fell on the waist when braking to avoid the car from west to east. [redacted], female, 67 years old. T12 lumbar vertebral compression fracture. Changzhou First Hospital for medical treatment. Tel: [redacted].
5	由北向南避让右侧同方向变道的轿车采取制动致车内一名女乘客78岁[redacted]受伤，伤情：头部血肿腰部挫伤在常州中医院治疗。伤者家属电话：[redacted]	A 78-year-old female passenger in the car, [redacted], got injured. The injuries: head hematoma and waist contusion were treated in Changzhou Hospital of Traditional Chinese Medicine. Family members of the injured Tel: [redacted].
6	[redacted]上行至上述时间地点时与轿车相擦	When [redacted] (bus driver) moved slowly to the above time and place, the bus sideswiped with the car.
6	[redacted]下行至上述时间地点时与轿车相擦	In particular, the bus sideswiped the car when [redacted] (bus driver) arrived at the above time and place.
6	[redacted]上行至上述时间地点时与轿车相擦	When [redacted] (bus driver) moved slowly to the above time and place, the bus sideswiped with the car.
6	[redacted]下行至上述时间地点时与轿车相擦	When [redacted] (bus driver) moved slowly to the above time and place, the bus sideswiped with the car.
6	[redacted]下行至上述时间地点时与轿车相擦，对方逃逸	[redacted] (bus driver) sideswiped the car when he arrived at the above time and place, and the other party fled.
7	由南向东与一辆由西向东的面包车相擦。致公交车无损，面包车左侧擦伤。	From south to east, it sideswipe a van from west to east. The bus was damaged and the left side of the van was scratched.
7	由东向西与左侧面包车相擦。公交车：左前受损，面包车：右后受损。	It sideswipe the left van from east to west. Bus: left front damaged, van: right rear damaged.
7	由东向西与一辆由西向东的货车相擦。致公交车左后角油漆擦伤，货车无损。	From east to west, it sideswipe a truck from west to east. The paint on the left rear corner of the bus was scratched, and the truck was damaged.
7	由西向东与一辆同方向右侧面包车相擦。致公交车右后侧边锁受损，面包车前保险杠擦伤。	From west to east, it sideswipe a van on the right in the same direction. As a result, the right rear side lock of the bus was damaged, and the front bumper of the van was scratched.
7	由东向西与一辆由北向西的面包车相擦。致公交车右侧车身油漆擦伤，面包车左前保险杠受损。	From east to west, it sideswipe a van from north to west. The paint on the right side of the bus was scratched, and the left front bumper of the van was damaged.

8	奔牛万家福239省道由北向南行驶追尾卡车	Benniu Wanjiafu Provincial Highway 239 runs from north to south and collides with a truck.
8	由南向北行驶至小河董家桥与一由北向南行驶的卡车相撞	Driving from south to north to Xiaohe Dongjiaqiao and collided with a truck travelling from north to south.
8	盛世名门由南向北行驶与一轿车相擦	The famous gate of the prosperous age is driving from south to north and sideswipe a car.
8	新冶路冶金厂地段由北向南行驶与轿车撞	The Xinye Road Metallurgical Plant was traveling from north to south and collided with a car.
8	吕汤路吕墅站由北向南行驶与一卡车擦。	Lushu Station, Lutang Road, traveled from north to south and was sideswipe with a truck.
9	路口左转弯，与一斑马线上过马路行人擦致使该行人受伤	Turning left at the intersection, it sideswipe with a pedestrian crossing the road on a zebra crossing, causing the pedestrian to be injured.
9	路口左转弯时，与一斑马线上行人擦致使行人受伤	When turning left at the intersection, it sideswipe with a pedestrian on a zebra crossing, causing the pedestrian to be injured.
9	行驶至此，左转弯时与一对方向小车擦	At this point, when making a left turn, it sideswipe a pair of directional trolleys.
9	行驶至竹林北路，追尾两辆小车，全责	Driving to Zhulin North Road, rear-end two cars, full responsibility.
9	行驶至此路口左转弯，与一对方向小车擦致使小车内乘员受伤	Driving to this intersection, turning left and sideswipe a pair of directional trolleys, causing injuries to the occupants in the trolley.
10	[redacted]下行至上述时间地点时与固定物相擦	When [redacted] (bus driver) arrived at the above time and place, he sideswipe the fixed object.
10	[redacted]下行至上述时间地点时与固定物相擦	When [redacted] (bus driver) arrived at the above time and place, it sideswipe the fixed object.
10	[redacted]下行至上述时间地点时与固定物相擦	When [redacted] (bus driver) goes down to the above time and place, it sideswipe the fixed object.
10	[redacted]上行至上述时间地点时与固定物相擦	When [redacted] (bus driver) arrives at the above time and place, it sideswipe the fixed object.
10	[redacted]下行至上述时间地点时与固定物相擦	When [redacted] (bus driver) goes down to the above time and place, it sideswipe the fixed object.
11	[redacted]于上述时间下行至广化街吊桥路路口时与残疾车相撞	[redacted] (bus driver) collided with a disabled vehicle when he descended to the intersection of Guanghua Street and Suspension Bridge Road at the above mentioned time.
11	[redacted]于上述时间下行至延陵路新世纪段时与轿车相擦	[redacted] (bus driver) collided with the car when he descended to the new century section of Yanling Road at the time mentioned above.
11	[redacted]于上述时间下行至桃园路德安桥段时与轿车相擦	[redacted] (bus driver) collided with the car when he descended to the Dean Bridge section of Taoyuan Road at the above time.
11	[redacted]于上述时间下行至延陵西路新世纪段时与轿车相擦	[redacted] (bus driver) collided with the car when he descended to the new century section of Yanling West Road at the time mentioned above.
11	[redacted]于上述时间下行至龙游南路三宝浜桥段时车内客伤	[redacted] (bus driver) got injured when he descended to the Sanbaobang Bridge section of Longyou South Road at the above time.
12	出厂时被地面翘出的钢板刮到水箱。	When leaving the factory, it was scraped to the water tank by the steel plate protruding from the ground.
12	加完气加气枪未拔起步将加气管拔断受损。	After the refilling, the refilling gun is not pulled out and the refilling pipe is pulled out and damaged.
12	修复区前溜刮擦外墙。	Slip and scrape the exterior wall before the restoration area.
12	收银后倒车时刮擦墙柱。致己车后尾灯受损。墙柱无损。	Scratching the wall post when reversing after checkout. Damaged rear taillights. Wall pillars are intact.
12	通过地道，车顶擦地道顶部。	Through the tunnel, the roof sideswipes the top of the tunnel.

13	顺园一村站停站时起步时一乘客要求下车，驾刹车一乘客摔倒受伤	At Shunyuan Yicun Station, a passenger asked to get off at the start of the stop and braked, and a passenger fell and got injured
13	民航服务中心站下客时开门门皮条带着乘客，乘客摔倒。	When getting off the passenger at the Civil Aviation Service Center, the door was opened with a leather strap carrying the passenger, and the passenger fell.
13	红梅公交中心站出站时急刹车内一乘客受伤，驾驶员带去看。	A passenger got injured when he braked suddenly when leaving the Hongmei Bus Center Station, and the driver took him to see it.
13	奥体中心站停站刹车一乘客受伤	One passenger injured after braking at Olympic Sports Center Station
13	停站下客后，关门夹伤乘客的脚。	After stopping to get off passengers, the door was closed and the passenger's feet were injured.
14	由北向南至科教城转盘时，因转弯角度小导致轮胎扎在人行道上，轮胎扎破。	When going from north to south to the turntable of the Science and Education City, the tire was stuck on the sidewalk due to the small turning angle, and the tire was punctured.
14	[redacted]于上述时间地点时倒溜与车辆相擦	At the above time and location, [redacted] (bus driver) slipped backwards and sideswiped the vehicle.
14	因道路施工颠簸致公交车前保险杠破裂。	The front bumper of the bus ruptured due to bumps in road construction.
14	因道路施工路面凹坑较大，车辆通过时颠簸与路基相刮。致公交车左后轮处油漆受损。	Due to the large pits in the road construction, the bumps and the roadbed are scraped when the vehicle passes. Damaged paint on the left rear wheel of the bus.
14	车辆前溜与前方卡车擦。	The vehicle slipped forward and sideswipe the truck in front.
15	行驶至此，被小车擦。	At this point, it was sideswiped by a car.
15	行驶至此，被小车擦左后尾。	At this point, the left rear tail was sideswiped by the car.
15	行驶至此，被一变道小车擦左前门。	At this point, the left front door was sideswiped by a lane-changing car.
15	行驶至此，被掉头小车擦	At this point, it was sideswiped by a U-turn car
15	行驶至此，被一小车擦左侧车身。	At this point, the left side body was sideswiped by a small car.

4 | XGBOOST

The XGBoost is a scalable tree boosting system, proposed by Chen and Guestrin (2016). It uses different split finding algorithms to improve the regularized learning of tree-based ensemble methods. We can denote the merged tabular data by $\mathscr{D} = \{(x_i, y_i)\}_{i=1}^{I}$, where x_i represents the vector of feature variables for the ith instance, and y_i represents its corresponding bus accident severity, which is the target variable that can have two or more classes or categories. Let $f(x_i)$ denote the XGBoost's prediction function on x_i, like many other tree-based ensemble methods, it is the sum of output from base learners, that is $\hat{y}_i = f(x_i) = \sum_m \phi_m(x_i)$, where $m = 1, \cdots, M$ is the index of base learners, ϕ_m is the mth base learner's prediction function, and the base learner is the classification and regression tree (Breiman *et al.*, 1984).

The XGBoost has a regularized loss function:

$$\mathscr{L}(f) = \sum_i l(y_i, \hat{y}_i) + \sum_m \Omega(\phi_m), \tag{3}$$

where $l(y_i, \hat{y}_i)$ is the differentiable convex loss function that measures the difference between the prediction $\hat{y}_i$ and the ground truth y_i, and $\Omega(\phi_m)$ is the regularizer that applies penalty to the model's complexity. As the accident severity variable in our data only contains two classes, we use the XGBoost to deal with two-class classification task so $l(y_i, \hat{y}_i)$ can be computed as follows

$$l(y_i, \hat{y}_i) = y_i \ln\{1 + e^{-\hat{y}_i}\} + (1 - y_i)\ln\{1 + e^{\hat{y}_i}\}, \tag{4}$$

and for base learner ϕ_m, the regularizer $\Omega(\phi_m)$ can be formulated as

$$\Omega(\phi_m) = \alpha\tau_m + \frac{1}{2}\lambda \parallel \varpi_m \parallel_2^2, \tag{5}$$

where α and λ are hyperparameters that control model complexity and regularization strength, τ_m is the number of leaves, ϖ_m is the vector of leaf weights, and $\parallel \cdot \parallel_2$ is the Euclidean norm.

The model is trained in an additive manner. Let $\hat{y}_i^{(n)}$ be the prediction of the ith instance at the nth iteration, then the model training can be performed by greedily adding f_n that most improves the following loss function

$$\begin{aligned} \mathscr{L}^{(n)} &= \sum_i l(y_i, \hat{y}_i^{(n-1)} + f_n(x_i)) + \Omega(f_n) \\ &\simeq \sum_i \left[g_i f_n(x_i) + \frac{1}{2}h_i f_n^2(x_i)\right] + \Omega(f_n), \end{aligned} \tag{6}$$

where $g_i = \partial l(y_i, \hat{y}_i^{(n-1)})/\partial \hat{y}_i^{(n-1)}$ and $h_i = \partial^2 l(y_i, \hat{y}_i^{(n-1)})/\partial(\hat{y}_i^{(n-1)})^2$.

Let $S_\varsigma = \{i | q(x_i) = \varsigma\}$ be the set of instances of leaf ς. Eq. (6) can then be rewritten as follows

$$\begin{aligned} \mathscr{L}^{(n)} &= \sum_i \left[g_i f_n(x_i) + \frac{1}{2}h_i f_n^2(x_i)\right] + \alpha\tau_m + \frac{1}{2}\lambda \sum_\varsigma (\varpi_m^{(\varsigma)})^2 \\ &= \sum_\varsigma \left[\sum_{i\in S_\varsigma} g_i \varpi_m^{(\varsigma)} + \frac{1}{2}\left[\sum_{i\in S_\varsigma} h_i + \lambda\right](\varpi_m^{(\varsigma)})^2\right] + \alpha\tau_m. \end{aligned} \tag{7}$$

For a fixed structure $q(x)$, the optimal weight $\varpi_m^{(\varsigma)*}$ of leaf ς is

$$\varpi_m^{(\varsigma)*} = -\frac{\sum_{i\in S_\varsigma} g_i}{\sum_{i\in S_\varsigma} h_i + \lambda}, \tag{8}$$

so the optimal loss is

$$\mathscr{L}^{(n)}(q) = -\frac{1}{2}\sum_{\varsigma}\frac{\left(\sum_{i\in S_\varsigma} g_i\right)^2}{\sum_{i\in S_\varsigma} h_i + \lambda} + \alpha\tau_m. \tag{9}$$

It is impossible to enumerate all the possible tree structures so a greedy algorithm can be used. In practice, after the split, we denote S_L and S_R the left and right nodes, where $S = S_L \cup S_R$. Then the loss reduction after the split can be computed as follows

$$\mathscr{L}_{\text{Split}} = \frac{1}{2}\left[\frac{\left(\sum_{i\in S_L} g_i\right)^2}{\sum_{i\in S_L} h_i + \lambda} + \frac{\left(\sum_{i\in S_R} g_i\right)^2}{\sum_{i\in S_R} h_i + \lambda} + \frac{\left(\sum_{i\in S} g_i\right)^2}{\sum_{i\in S} h_i + \lambda}\right] - \alpha. \tag{10}$$

5 | HYPERPARAMETER TUNING AND MODEL BENCHMARKING

Table 3 summarizes the parameter search space used in the grid search procedure during cross-validation. This approach systematically evaluates combinations of hyperparameters to identify the optimal configuration, ensuring robust model performance and minimizing the risk of overfitting.

TABLE 3 Hyperparameter tuning of the XGBoost and benchmarked algorithms.

Model	Hyperparameter setting
Logistic regression	"solver": "liblinear"
KNN	"n_neighbors": [10, 20, 30, 40, 50],<br>"algorithm": ["ball_tree", "kd_tree"],<br>"leaf_size": [20, 30, 40, 50],<br>"weights": ["uniform", "distance"],<br>"metric": ["euclidean", "manhattan", "minkowski"]
Random forest	"max_depth": [10, 25, 50],<br>"min_samples_leaf": [1, 5, 10],<br>"min_samples_split": [2, 5, 10],<br>"n_estimators": [100, 200, 500]
AdaBoost	"learning_rate": [0.001, 0.01, 0.05, 0.1, 0.15],<br>"n_estimators": [100, 200, 500]
GBDT	"learning_rate": [0.001, 0.01, 0.05, 0.1, 0.15],<br>"n_estimators": [100, 200, 500]
XGBoost	"learning_rate": [0.001, 0.01, 0.05, 0.1, 0.15],<br>"max_depth": [10, 25, 50],<br>"min_child_weight": [2, 3, 4],<br>"gamma": [0.0, 0.1, 0.2 , 0.3, 0.4],<br>"colsample_bytree": [0.3, 0.4, 0.5, 0.7]
MLP	"hidden_layer_sizes": [(50, 50, 50), (100, 100, 100),(200, 200, 200), (100,)],<br>"activation": ["sigmoid", "relu", "tanh"],<br>"solver": ["adam", "lbfgs", "sgd"],<br>"alpha": [0.001, 0.01, 0.05, 0.1, 0.15],<br>"learning_rate": ["constant", "adaptive"]

6 | THEORETICAL AXIOMS OF SHAP

SHAP (SHapley Additive exPlanations) (Lundberg and Lee, 2017) possesses several desirable theoretical properties that underpin its robustness and credibility as a model-agnostic interpretability technique. Specifically, it satisfies three key axioms derived from cooperative game theory: (i) local accuracy (also known as efficiency), (ii) missingness, and (iii) consistency. Together, these properties ensure that SHAP values yield meaningful and mathematically grounded attributions of feature importance.

The local accuracy property guarantees that the sum of all feature attributions equals the difference between the model's prediction for a specific instance and its expected output. Formally, for a predictive model f at input x, the SHAP values $\xi_j(f, x)$ for each feature j satisfy:

$$f(x) = \xi_0(f, x) + \sum_j \xi_j(f, x), \tag{11}$$

where $\xi_0(f, x) = \mathbb{E}[f(x)]$ denotes the model's expected prediction. This property ensures that SHAP explanations faithfully decompose the model output into additive feature contributions.

The missingness property stipulates that if a feature is absent from the model or exerts no influence on the prediction, its attribution should be zero. Formally, if including feature j in coalition W does not alter the prediction, i.e., $f_x(W \cup j) = f_x(W)$, then $\xi_j(f, x) = 0$. This guarantees that SHAP does not assign importance to irrelevant features.

Finally, the consistency property requires that if a model is modified such that the marginal contribution of a feature increases (or remains constant) across all coalitions, its SHAP value should not decrease. More formally, for two models f and φ, if for all coalitions $W \subseteq \mathscr{W} \setminus j$,

$$f_x(W \cup j) - f_x(W) \geq \varphi_x(W \cup j) - \varphi_x(W), \tag{12}$$

then $\xi_j(f, x) \geq \xi_j(\varphi, x)$. This property ensures logical coherence and fairness when comparing feature attributions across models or iterations.

REFERENCES

Airoldi, E. M. and Bischof, J. M. (2016). Improving and evaluating topic models and other models of text. *Journal of the American Statistical Association*, 111, 1381–1412.

Breiman, L., Friedman, J. H., Olshen, R. A. and Stone, C. J. (1984). *Classification and regression trees*. : Routledge.

Chen, T. and Guestrin, C. (2016). XGBoost: A scalable tree boosting system. *Proceedings of the 22nd ACM SIGKDD International Conference on Knowledge Discovery and Data Mining*, 8, 785–794.

Lundberg, S. M. and Lee, S. I. (2017). A unified approach to interpreting model predictions. *Proceedings of the 31st International Conference on Neural Information Processing Systems*, 12, 4768–4777.

Mimno, D., Wallach, H. M., Talley, E., Leenders, M. and McCallum, A. (2011). Optimizing semantic coherence in topic models. *Proceedings of the Conference on Empirical Methods in Natural Language Processing*, 1, 262–272.

Roberts, M. E., Stewart, B. M. and Tingley, D. (2019). STM: An R package for structural topic models. *Journal of Statistical Software*, 91, 1–40.

□
